# Identifying female pelvic cancer survivors with low levels of physical activity after radiotherapy: women with fecal and urinary leakage need additional support

**DOI:** 10.1007/s00520-019-05033-3

**Published:** 2019-10-22

**Authors:** Anna Lindgren, G. Dunberger, G. Steineck, K. Bergmark, A. Enblom

**Affiliations:** 1 County Council of Östergötland and Department of Medical and Health Sciences, Division of Physiotherapy, Linköping University, SE-58183 Linköping, Sweden; 2 Department of Health Care Sciences, Ersta Sköndal University College, Stockholm, Sweden; 3 Department of Oncology-Pathology, Division of Clinical Cancer Epidemiology, Karolinska Institute, Stockholm, Sweden; 4 Department of Clinical Sciences, Division of Clinical Cancer Epidemiology, Sahlgrenska Academy, Gothenburg University, Gothenburg, Sweden; 5 County Council of Östergötland, Activity and Health and Division of Coordinated Cancer Evaluation, Department of Medical and Health Sciences, Linköping University, Linköping, Sweden

**Keywords:** Colorectal cancer, Fecal leakage, Gynecological cancer, Incontinence, Physical activity, Urinary leakage

## Abstract

**Objective:**

To investigate the frequency of physical activity among female pelvic cancer survivors (i.e., gynecological, rectal, and anal cancer survivors) and to investigate if survivors who practiced physical activity less than once a week differed from survivors practicing physical activity at least once a week with respect to urinary and fecal leakage, clinical and sociodemographic characteristics, quality of life (QoL), and depressed and anxious mood.

**Methods:**

Female pelvic cancer survivors (*n* = 578, mean age 64 years) answered a questionnaire 6–48 months after radiotherapy. A multivariable regression model analyzed factors covarying with frequency of physical activity. We compared QoL and depressed and anxious mood between women practicing physical activity at least or less than once a week.

**Results:**

Of 568 women delivering data, 186 (33%) practiced physical activity less than once a week while 382 (67%) practiced physical activity at least weekly. Women who leaked a large or all volume of stools (*p* = 0.01), had just elementary school level of education (*p* < 0.001), smokers (*p* = 0.049), or had lymphedema without receiving lymphedema treatment (*p* = 0.030) were more likely to practice physical activity less than weekly (50%, 45%, 45%, and 37%, respectively) compared with other women. Women practicing physical activity at least weekly reported better QoL (*p* < 0.001) and lower frequency of depressed mood (*p* = 0.044) compared with the others.

**Conclusions:**

Female cancer survivors experiencing fecal leakage were less likely to practice weekly physical activity than survivors without leakage. The survivors practicing weekly physical activity experienced better QoL and experienced depressed mood less frequently than the others.

## Introduction

Several studies have found that physical activity results in health benefits for cancer survivors [1], but few studies [2–4] have investigated the adherence to and potential barriers to physical activity in female cancer survivors after pelvic radiotherapy. Physical activity means bodily movement produced by skeletal muscles that require energy expenditure [5]. Previous studies observed that physical activity in cancer survivors can produce a variety of beneficial health effects [1, 6] and has a relationship with reduced risk of recurrence and mortality in cancer, for example, ovarian cancer, breast cancer, colon cancer, and rectal cancer [7, 8]. A risk reduction of 37% in cancer-specific mortality was reported in a review comparing patients who were most physically active with those who were least physically active based on 26 studies of breast, colorectal, and prostate cancer [9].

Reports of increasing numbers of observed health benefits of physical activity in cancer survivors [1, 6] have led to guidelines regarding physical activity in cancer survivors [10], underscoring the importance of exercise in cancer rehabilitation. In Sweden [11] and other countries [10], guidelines recommend at least 150 min of moderate intensity physical activity, or 75 min of vigorous physical activity, weekly. Despite strong evidence for the health benefits of physical activity in cancer survivors [1, 6], many survivors do not adhere to the guidelines [3, 4, 12, 13]. Cancer survivors in general tend to decrease their level of physical activity compared with the level pre-cancer treatment [14]. With pelvic cancer, survival is meant in the present study gynecological, rectal, and anal cancer survivors. Colorectal cancer survivors reported a low frequency of physical activity and high volume of sedentary time after cancer treatment [15]. Thirteen percent of gynecological cancer survivors stated that they never practiced physical activity after cancer treatment [2].

Radiotherapy is an important life-saving part of cancer treatment. However, radiotherapy induces an inflammatory process that can lead to fibrosis and thereby reduces elasticity and activation of the pelvic floor muscles. In addition, radiotherapy often induces nerve damages and reduces blood flow within the muscular tissues, which further may complicate muscular activation and may cause atrophy [16]. Symptoms that occur or persist for more than 3 months after radiotherapy are referred to as late side effects. These long-term unwanted pathophysiological consequences of radiotherapy [17, 18] often lead to urinary and fecal leakage [2, 18–21]. Fecal and urinary leakage is one of the most serious late consequences of pelvic radiotherapy that complicates life and aging [19–21]. Fecal leakage is also defined as the leakage syndrome [18]. Our [18] and others’ [16, 17] data indicate treatment-induced late consequences may be lifelong.

In a previous qualitative study, female pelvic cancer survivors experienced leakage to be a barrier for practicing physical activity, especially if there was no bathroom nearby. Lack of physical activity decreased their psychological energy and physical fitness [22]. Based on observations from non-cancer female populations [23], it is plausible that leakage may be a barrier for pelvic cancer survivors keeping them from practicing physical activity. However, according to our database searches, no previous quantitative studies have explored the frequency of physical activity in relation to urinary or fecal leakage among female pelvic cancer survivors (www.ncbi.nlm.nih.gov/pubmed, search terms (physical activity) AND cancer AND (incontinence OR leakage), date 12.11.18).

The objective of our study was to investigate frequency of physical activity among female pelvic cancer survivors after radiotherapy and to investigate if survivors who practiced physical activity less than once a week differed from survivors practicing physical activity at least once a week regarding urinary and fecal leakage, clinical and sociodemographic characteristics, quality of life, and depressed and anxious mood.

## Methods

### Setting and inclusion

The setting was outpatient in southwest Sweden. By examining the electronic records, a nurse, at a rehabilitation unit at a university hospital, identified female pelvic cancer survivors who had received pelvic radiotherapy between 2007 and 2016. The rehabilitation unit also received referrals from within and outside the university hospital. The identified or referred pelvic cancer survivors were screened for the study criteria. Inclusion criteria: Received external pelvic radiotherapy with or without intracavity brachytherapy for gynecological, rectal, or anal cancer 6–48 months ago. Exclusion criteria: Ongoing cancer, physical, psychological, or linguistic (not understanding Swedish) issues that made it difficult for them to give informed consent.

An information letter offered a follow-up visit unit for support regarding radiotherapy-induced consequences. To document potential consequences, the information letter asked the survivors to answer a study-specific questionnaire, attached with the information letter, data from which was used in this study. The regional ethics committee approved the study (Gothenburg 686-10), which adhered to the declaration of Helsinki.

### The study-specific questionnaire

The study-specific questionnaire (Appendix) was developed and satisfactorily validated according to clinimetric methodology [2, 24], frequently applied when studying cancer survivors [2, 20, 25]. Details of the validation procedure have been previously reported [2].

#### Sociodemographic and clinical characteristics

The women reported sociodemographic and clinical characteristics and a nurse collected data on previous cancer diagnoses and radiotherapy data from the medical records.

#### Frequency of physical activity

The women answered the question: “Do you practice physical activity?” (Seven categories ranging from “No to “Yes, at least once a day”). The question correlated well to the Swedish Board of Welfare and Health indicator question regarding moderate level physical activity (Spearman’s correlation coefficient, *r*_s_, 0.556, *n* = 449), which is valid compared with accelerometer-measured activity [26]. The women were not given any definition of physical activity before answering the question but were free to interpret the question from their own perception of physical activity.

#### Urinary and fecal leakage

The questionnaire asked about urinary leakage, one example being: “Have you been wetting yourself because you could not reach the toilet in time within the past six months?” (Six categories, “No” to “Yes, at least once a day”), and “How large volume do you leak? (Four categories, “Not applicable,” to “All bladder volume”). The questionnaire asked about fecal leakage, one example being: “Have you leaked stools because you could not reach the toilet in time, within the past six months?” (Six categories, “No” to “Yes, at least daily”), and “How large volume do you leak? (Four categories, “Not applicable, I do not leak stool” to “All volume of stools”).

#### Quality of life and depressed and anxious mood

The women graded their experienced quality of life and their level of depressed and anxious mood on 7-point numeric rating scales: “How has your quality of life been the last six months? (“No quality of life at all” to “Best possible quality of life”), “Have you felt low or depressed within the past six months?”, and “Have you felt anxious within the past six months?” (“Never” to “All the time”). These questions demonstrated high co-variation and consistency with established instruments [27].

### Statistical analysis

We calculated descriptive data: number (*n*) and proportions (percent) for all variables, mean value with standard deviation (± 1 SD) for continuous variables, and median (md) with 25th and 75th percentiles (IQR) for ordinal variables. We categorized the physical activity data into two groups: “Practiced physical activity at least once a week” (i.e., “Yes, at least once a week,” “Yes, at least three times a week,” and “Yes, at least once a day”) and “Practiced physical activity less than once a week” (“No,” “Yes, occasionally,” “Yes, at least once a month”), based on guidelines [9] that recommend weekly physical activity. Fisher’s exact test compared subgroups with different characteristics regarding urinary and fecal leakage, and sociodemographic and clinical variables, presented as relative risks (RR) for practicing physical activity less than once a week, with 95% confidence intervals (CI). The reference category was defined as the category with the lowest proportion not practicing physical activity at least once a week. We selected volume of fecal and urinary leakage and other possible sociodemographic and clinical variables (all variables seen in Table 4 resulting in a *p* value of < 0.10, according to the univariable analysis) explaining the variation of physical activity with a multivariable generalized linear model, and generated relative risks using binomial distribution and log link. A response analysis was also made to ensure that the loss in the multivariable analysis did not affect its results. We compared the physical activity groups using Mann-Whitney *U* test regarding quality of life, depressed mood, and anxious mood (ordinal variables). The analyses were performed in Statistical Package of Social Science (SPSS) for Windows, version 24.0, and the significance level was 5%.

## Results

### The participating women

**Fig. 1:**
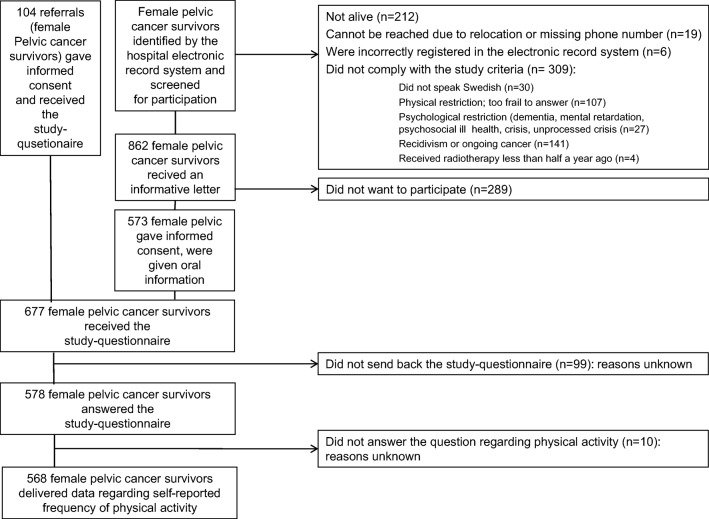
Flowchart of the screened, included and responding female pelvic cancer survivors

Of the 677 female pelvic cancer survivors who were included and received the study questionnaire, 99 did not answer the questionnaire, while 578 answered (response rate 85%) and 568 (98%) of the answering women provided data regarding physical activity (Fig. 1). Mean age was 64 years. Table 1 presents clinical and demographic characteristics.

**Table 1:** Characteristics of the study participants

Variable	Total study group of female pelvic cancer survivors, *n* = 578
Cancer type, *n* (%)	
Gynecological cancer	367 (63)
Rectal cancer	131 (23)
Anal cancer	77 (13)
Other	3 (1)
Age in years, mean ± SD	*n* = 577 64 ± 12.9
Years since radiotherapy, mean ± SD	*n* = 576 2.7 ± 3.4
Cancer treatment, *n* (%)	*n* = 577
External radiotherapy, only	131 (23)
Brachytherapy, only	1 (0.2)
External radiotherapy and brachytherapy	20 (3)
External radiotherapy, brachytherapy and surgery	178 (31)
External radiotherapy and surgery	243 (42)
Brachytherapy and surgery	4 (0.6)
Marital status, *n* (%)	
Married or living with a partner	386 (67)
Widow	66 (11)
Has a partner but lives alone	28 (5)
Single	98 (17)
Education level, *n* (%)	*n* = 569
Elementary school	171 (30)
Secondary school	211 (36)
Collage/university	187 (32)
Employment status, *n* (%)	*n* = 573
Student	4 (0.6)
Unemployed job seeker	11 (2)
Employed, n (%)	161 (28)
Home maker	4 (0.6)
On sick leave	48 (8)
Disability pension	31 (5)
Retired	314 (54)
Resident, *n* (%)	*n* = 577
On the countryside	108 (19)
Small or medium-sized city	297 (51)
In a big city	172 (30)
Smoking, *n* (%)	*n* = 555
Yes	66 (11)
No	489 (85)

*n* (number) and proportion (%) of women are presented, *n* delivering data is presented in case of missing data. SD = standard deviation

### Frequency of physical activity

Of 568 women provided data regarding physical activity; 68 (12%) never practiced physical activity, 101 (18%) occasionally (at single occasions), 17 (3%) at least once a month, 160 (28%) at least once a week, 160 (23%) at least three times a week, and 92 (16%) practiced physical activity at least once a day. Accordingly, 383 (67%) practiced physical activity at least once a week while 186 (33%) practiced physical activity less than once a week.

### Physical activity in women with or without urinary leakage

Of the 27 women who reported daily urinary leakage in case of urgency, 52% practiced physical activity less than once a week, compared with 29% of the 310 women who reported no urinary leakage when answering a question regarding frequency of urinary leakage (*p* = 0.005, Table 2). Of the 74 women who could keep urine less than a minute in case of urgency, 55% practiced physical activity less than once a week, compared with 26% of the 76 women who could keep urine more than 30 min (*p* < 0.001, Table 2). Furthermore, of the 74 women who reported a moderate to all volume of leakage, 32% practiced physical activity less than once a week, compared with 27% of the 266 women who reported no volume of urinary leakage when answering a question regarding volume of urinary leakage (*p* = 0.04, crude models, Table 4). However, when adding the other independent variables into the analysis, for example, volume of fecal leakage, the relationship between urinary leakage and the dependent variable frequency of practicing physical activity was not significant (*p* = 0.105, adjusted models, Table 4). According to the response analysis, the loss in the multivariable analysis did not affect its results.

**Table 2:** Frequency of physical activity in female pelvic cancer survivors with various severities of urinary leakage

Variable	Practicing physical activity at least once a week, *n* = 382	Practicing physical activity less than once a week, *n* = 186	Relative risk, 95% CI, *p* value, *n* = 568
Frequency of leakage in case of urgency, *n* (%)	*n* = 374	*n* = 182	
No	220/310 (71)	90/310 (29)	Ref.
Occasionally	105/162 (65)	57/162 (35)	1.21, 0.14–0.92, 0.166
At least once a month	10/20 (50)	10/20 (50)	1.72, 1.07–2.76, 0.024*
At least once a week	17/24 (70)	7/24 (29)	1.0, 0.53–1.92, 0.989
At least three times a week	9/13 (69)	4/13 (31)	1.06, 0.46–2.44, 0.891
At least once a day	13/27 (48)	14/27 (52)	1.79, 1.19–2.67, 0.005*
Volume of leakage, *n* (%)	*n* = 371	*n* = 177	
No leakage	193/262 (74)	69/262 (26)	Ref.
Small volume of urine	132/207 (63)	75/207 (36)	1.38, 1.05–1.80, 0.021*
Large volume of urine	37/63 (59)	26/63 (41)	1.57, 1.10–2.24, 0.014*
All volume of urine	5/11 (45)	6/11 (55)	2.10, 1.16–3.69, 0.014*
Capacity to hold urine before leakage in case of urgency, *n* (%)	*n* = 366	*n* = 178	
At least 30 min	56/76 (74)	20/76 (26)	Ref.
10–30 min	89/124 (72)	35/124 (28)	1.07, 0.67–1.72, 0.77
5–10 min	92/127 (72)	35/127 (28)	1.05, 0.65–1.68, 0.847
1–5 min	96/143 (67)	47/143 (33)	1.25, 0.80–1.95, 0.326
Less than 1 min	33/74 (45)	41/74 (55)	2.11, 1.37–3.23, < 0.001*

*n* (number) and proportion (%) of women are presented, *n* delivering data is presented in case of missing data. Ref. = Reference category, relative risk 1.0; the category free from or with lowest severity of leakage. CI = 95% confidence interval, *statistically significant difference. The time frame of the questions was the past 6 months

### Physical activity in women with or without fecal leakage

Of the 13 women who reported daily fecal leakage in case of urgency, 62% practiced physical activity less than once a week, compared with 30% of the 223 women with no fecal leakage when answering a question regarding frequency of fecal leakage (*p* = 0.004). Of the 98 women who reported fecal leakage without forewarning anytime within the preceding 6 months, 50% practiced physical activity less than once a week (*p* < 0.001), compared with 30% of the 448 women who did not report any fecal leakage without forewarning when answering a question regarding volume of fecal leakage (Table 3). Of the 54 women who reported a large or all volume of fecal leakage, 50% practiced physical activity less than once a week compared with 31% of the 388 women with no volume of fecal leakage or just soiling when answering a question regarding volume of fecal leakage (*p* = 0.002, crude models, Table 4). When adding the other independent variables into the analysis, the relationship between fecal leakage and the dependent variable frequency of practicing physical was still significant (*p* = 0.105, adjusted models, Table 4).

**Table 3:** Frequency of physical activity in female pelvic cancer survivors with various severities of fecal leakage

Variable	Practicing physical activity at least once a week, *n* = 382	Practicing physical activity less than once a week, *n* = 186	Relative risk, 95% CI, *p* value, *n* = 568
Frequency of leakage in case of urgency, *n* (%)	*n* = 365	*n* = 170	
No	155/223 (68)	68/223 (30)	Ref.
Occasionally	135/194 (70)	59/194 (30)	1.00, 0.75–1.33, 0.986
At least once a month	26/40 (65)	14/40 (35)	1.15, 0.72–1.83, 0.563
At least once a week	33/47 (70)	14/47 (30)	0.98, 0.60–1.58, 0.924
At least three times a week	11/18 (61)	7/18 (39)	1.28, 0.69–2.35, 0.436
At least once a day	5/13 (39)	8/13 (62)	2.02, 1.26–3.24, 0.004*
Volume of leakage, *n* (%)	*n* = 360	*n* = 172	
No leakage	156/223 (70)	67/223 (30)	Ref.
Just soiling	103/147 (70)	44/147 (30)	1.0, 0.72–1.37, 0.982
Small volume of stools	74/110 (67)	36/110 (33)	1.1, 0.78–1.52, 0.616
Large volume of stools	20/41 (49)	21/41 (51)	1.70, 1.19–2.44, 0.004*
All stools	7/11 (64)	4/11 (36)	1.21, 0.54–2.71, 0643
Leakage of all stools without forewarning, *n* (%)	*n* = 364	n = 177	
No	315/448 (70)	133/448 (30)	Ref.
Yes	49/98 (50)	49/98 (50)	1.68, 1.32–2.15**, <** 0.001*

*n* (number) and proportion (%) of women are presented, *n* delivering data is presented in case of missing data. Ref. = Reference category, relative risk 1.0; the category free from leakage. CI = 95% confidence interval, *statistically significant difference. The time frame of the questions was the past 6 months

**Table 4:** Frequency of physical activity in subgroups of female pelvic cancer survivors with or without fecal or urinary leakage and with a variety of sociodemographic and clinical characteristics

Variable	Practicing physical activity at least once a week, *n* = 382	Practicing physical activity less than once a week, *n* = 186	Relative risk, 95% CI, *p* value univariable analyses (crude models), *n* = 568	Relative risk, 95% CI, *p* value multivariable analysis (adjusted models), *n* = 492
Urinary leakage, *n* (%)	*n* = 369	*n* = 178	*n* = 547	
No leakage	195/266 (73)	71/266 (27)	Ref.	Ref.
Small leakage	132/207 (64)	75/207 (36)	1.36, 1.04–1.78, 0.026*	1.17, 0.89–1.54, 0.261
Moderate to large, i.e., moderate, large, or all volume	42/74 (57)	32/74 (43)	1.62, 1.17–2.25, 0.04*	1.33, 0.942–1.889, 0.105
Fecal leakage, *n* (%)	*n* = 369	*n* = 183	*n* = 552	
No leakage, i.e., no leakage or just soiling	268/388 (69)	120/388 (31)	Ref.	Ref.
Small leakage	74/110 (67)	36/110 (33)	1.06, 0.78–1.44, 0.72	1.02, 0.75–1.38, 0.91
Large volume, i.e., large or all volume	27/54 (50)	27/54 (50)	1.62, 1.19–2.19, 0.002*	1.54, 1.11–2.15, 0.01*
Cancer type, *n* (%)				
Gynecological cancer	230/359 (64)	129/359 (36)	1.32, 1.02–1.71, 0.034*	1.26, 0.96–1.65, 0.097
Not gynecological cancer	152/209 (73)	57/209 (27)	Ref.	Ref.
Age in years, *n* (%)	*n* = 382	*n* = 183		
24–45	39/59 (66)	20/59 (34)	1.08, 0.71–1.62, 0.710	Not applicable^5^
46–65	153/223 (69)	70/223 (31)	Ref.	
66–94	190/286 (66)	96/286 (34)	1.07, 0.83–1.38, 0.604	Not applicable^5^
Year since radiotherapy, *n* (%)	*n* = 381	*n* = 186		
Less than 1 year ago	151/227 (67)	76/227 (33)	1.13, 0.66–1.91, 0.66	Not applicable^5^
1–5 years ago	204/303 (67)	99/303 (33)	1.10, 0.65–1.85, 0.723	Not applicable^5^
More than 5 years ago	26/37 (70)	11/37 (30)	Ref.	
Cancer treatment, *n* (%)	*n* = 258	*n* = 114		
External radiotherapy, only	79/130 (61)	51/130 (39)	1.24, 0.89–1.73, 0.20	Not applicable^5^
External radiotherapy and surgery	174/237 (73)	63/237 (27)	Ref.	
Brachytherapy and surgery	5/5 (100)	0/5 (0)	Not applicable^1^	
Marital status**,***n* (%)	*n* = 382	*n* = 186		
Married, living together, or has a partner	285/408 (70)	123/408 (30)	Ref.	Ref.
Widow or single	97/160 (61)	63/160 (39)	1.31 1.02–1.66, 0.031*	1.24, 0.97–1.60, 0.091
Education level, *n* (%)	*n* = 378	*n* = 183		
Elementary school	90/164 (55)	74/164 (45)	1.64, 1.30–2.07, < 0.001*	1.62, 1.27–2.01, < 0.001*
Secondary school, college or university	288/397 (73)	109/397 (27)	Ref.	Ref.
Employment status, *n* (%)	*n* = 379	*n* = 184		
Unemployed job seeker	3/11 (27)	8/11 (73)	2.32, 1.52–3.56, < 0.001*	Not applicable^1^
Employed, housewife/man, or student	116/169 (69)	53/169 (31)	Ref.	
On sick leave	30/48 (63)	18/48 (37)	1.20, 0.78–1.83, 0.413	Not applicable^5^
Retired due to age or disability	230/335 (69)	105/335 (31)	1.0, 0.76–1.31, 0.999	Not applicable^5^
Resident, *n* (%)	*n* = 381	*n* = 186		
On the countryside	65/106 (61)	41/106 (39)	1.23, 0.93–1.62, 0.141	Not applicable^5^
In a small or big city	316/461 (69)	145/461 (31)	Ref.	
Smoking, *n* (%)	*n* = 370	*n* = 177		
Yes	36/66 (55)	30/66 (45)	1.49, 1.10–2.00, 0.009*	1.34, 1.00–1.79, 0.049*
No	334/481 (69)	147/481 (31)	Ref.	Ref.
Weight change^3^, *n* (%)	*n* = 337	*n* = 151		
No	118/164 (72)	46/164 (28)	Ref.	
Yes, weight gain	81/119 (68)	38/119 (32)	1.13, 0.82–1.56, 0.453	Not applicable^5^
Yes, weight loss	138/205 (67)	67/205 (33)	1.27, 0.93–1.74, 0.131	Not applicable^5^
Pain in the abdomen^2^**,***n* (%)	*n* = 370	*n* = 184		
Less than once a day	345/509 (68)	164/509 (32)	Ref.	Ref.
At least once a day	25/45 (56)	20/45 (44)	1.38, 0.97–1.96, 0.072	1.30, 0.89–1.904, 0.17
Lymphedema diagnose and treatment, *n* (%)	*n* = 386	*n* = 174		
No lymphedema diagnose	269/402 (67)	133/402 (33)	2.22, 1.11–4.46, 0.025*	2.02, 1.02–4.02, 0.045*
Lymphedema diagnose but has not received any treatment	59/93 (63)	34/93 (37)	2.45, 1.18–5.11, 0.017*	2.25, 1.08–4.67, 0.030*
Lymphedema diagnose and received treatment^4^	40/47 (85)	7/47 (15)	Ref.	Ref.

*n* (number) and proportion (%) of women are presented, *n* delivering data is presented in case of missing data. Ref. = Reference category, relative risk 1.0; the category with the lowest proportion practicing physical activity less than once a week. CI = 95% confidence interval, *statistically significant difference. ^1^Due to low *n*; ^2^Within the last 6 months; ^3^Compared with weight before cancer treatment; ^4^Treated by physiotherapist, nurse, or lymph therapist. ^5^Not included in the multivarable analysis due to *p* was not < 0.10 in the univariable analysis

### Physical activity in women with different sociodemographic and clinical characteristics

According to the multivariable analysis, widows or single women, women who had just an elementary school level of education, smokers, and women with lymphedema without receiving lymphedema treatment were more likely to practice physical activity less often than once a week compared with the other women.

### Physical activity in relation to quality of life and depressed and anxious mood

**Fig. 2:**
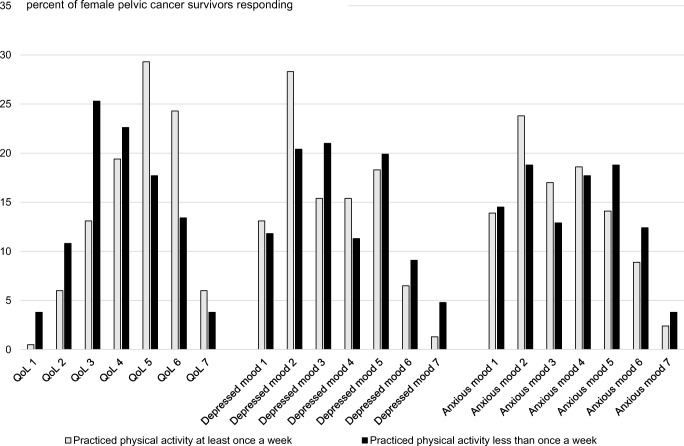
Grading of QoL (Quality of Life: 1; No quality of life to 7; Best possible) and frequency of depressed and anxious mood (1; Never to 7; All the time) in female pelvic cancer survivors practicing physical activity at least once a week or less than once a week. Of 578 women delivering physical activity data, 558 women delivered data on QoL, 559 on depressed mood, and 561 on anxious mood

The women practicing physical activity at least once a week reported better quality of life (md 5, IQR 4–6) and lower frequency of depressed mood (md 3, IQR 2–5) compared with women practicing physical activity less than once a week (quality of life: md 4, IQR 3–5, *p* < 0.001, depressed mood: md 3, IQR 2–5, *p* = 0.044). The observed tendency for women practicing physical activity at least once a week to experience anxious mood less frequently was not statistically significant (md 3, IQR 2–5 versus md 4, IQR 2–5, *p* = 0.071) (Fig. 2).

## Discussion

In summary, we found that one-third of female pelvic cancer survivors practiced physical activity less than once a week. Female cancer survivors experiencing a large volume of fecal leakage were less likely to practice physical activity compared with survivors without leakage and a clear similar trend was seen among those experiencing urinary leakage. Cancer survivors who had just an elementary school level of education, smokers, and survivors diagnosed with lymphedema without receiving lymphedema treatment practiced less physical activity than others. Survivors practicing physical activity at least weekly experienced better quality of life and less frequent experienced depressed mood than others.

The observation that one-third of the female pelvic cancer survivors practiced physical activity less than weekly tells us that these women may need more support in leading them to practice physical activity and even to help them understand the beneficial health effects of physical activity [1]. The frequency of physical activity in this third of the women was much lower than the recommended level of weekly physical activity for all survivors [10]. Several studies report low adherence to the physical activity recommendations [3, 4, 15, 28]. Almost all (96%) of 285,825 cancer survivors in general did not meet recommended physical activity guidelines 5 years after their cancer diagnosis [28]. One-third of 600 colorectal cancer survivors met physical activity guidelines while almost half were completely sedentary [3], in line with other observations [15]. Forbes and co-workers reported that 42% of breast, prostate, and colorectal cancer survivors (*n* = 741) met physical activity guidelines, with no differences among the cancer types [4]. However, our observations on female pelvic cancer survivors indicate that female cancer survivors seem to adhere to recommendations of weekly physical activity to a lower extent than male pelvic cancer survivors and other types of female cancer survivors. Prostate cancer survivors were more likely to meet physical activity guidelines (30%) than gynecological pelvic cancer survivors (12%) [29]. Furthermore, Irwin and co-workers reported that 72% of 1223 breast cancer survivors, i.e., female cancer survivors not exposed to the leakage syndrome, practiced the recommended ≥ 150 min a week of moderate to vigorous intensity physical activity [13].

It is well known from non-cancer populations that urinary [30] or fecal [31] leakage is a barrier for physical activity. Our study confirmed that pelvic cancer survivors who experienced fecal leakage also practiced less physical activity compared with others. According to the univariable analysis, survivors experiencing urinary leakage practiced less physical activity than women not experiencing urinary leakage. However, in the multivariable analysis, fecal leakage explained a greater part of the variation in frequency of physical activity. Women with urinary leakage were more likely to also experience fecal leakage (35%) than women without urinary leakage (24%). Thus, urinary leakage may still be a contributing factor to a low level of physical activity, but fecal leakage was a stronger predictive variable. In non-cancer Americans aged 20–85 years (*n* = 2565), higher severity of fecal incontinence was associated with less frequent objectively measured moderate to vigorous physical activity [31]. Gynecological cancer survivors experiencing emptying of all stools without forewarning had a tendency to be more likely to practice physical activity less than weekly (63%) compared with those without that experience (74%) [20]. In non-cancer women (*n* = 41,000), a quarter of elderly women and a third of mid-age women avoided vigorous physical activity because of urinary leakage [32]. Furthermore, the proportion perceiving that urinary leakage was a barrier to physical activity ranged from 9% in women experiencing slight leakage severity to 85% in women experiencing severe leakage, in *n* = 3364 non-cancer women, aged 18 to 60 years [33]. Our study design did not allow us to reveal if it is a causal relation or not between the leakage syndrome and the reduced level of physical activity. Health care professionals may hypothesize that lack of physical activity would cause weaker pelvic floor muscles and thus induce leakage. However, no association between physical activity level and pelvic floor muscle strength and endurance has been reported among non-cancer women (*n* = 58) [34]. Based on previous findings [20, 22, 30–34], it seems more plausible that the leakage induced reduced physical activity.

The findings that survivors who smoke and/or are less well educated practiced less physical activity are in line with findings among non-cancer general populations [35–37] and among cancer survivors participating in rehabilitation groups [29]. We observed that those diagnosed with lymphedema who received lymphedema treatment practiced more physical activity than the others, even more than women without lymphedema. The fact that rehabilitation professionals probably had encouraged physical activity [10], particularly among survivors with lymphedema, may explain this. Physical activity may improve both objective and subjective parameters of secondary lymphedema and is an important part of lymphedema-treatment [38].

Not surprisingly [33], pelvic cancer survivors practicing physical activity at least weekly reported better quality of life and less often depressed mood than women practicing less physical activity, which seems to be an important finding from our routine-care study context, in light of the great health benefits of physical activity reported in reviews [1, 6]. We have not found any study reporting the relationship between low physical activity and low quality of life among female pelvic cancer survivors suffering from fecal or urinary leakage. However, among breast cancer survivors, increased physical activity reduced clinician-rated depressed mood and improved quality of life [39]. Furthermore, practicing physical activity was related to reduce depressed mood in non-cancer general populations [40, 41]. In a recent study of 1.2 million Americans, individuals who exercise reported 1.49 fewer days of poor mental health within the previous month compared with those who did not exercise [40]. A meta-analysis (92 studies, total *n* = 4310) found a moderate effect of physical activity for reducing depression [41]. Finally, in non-cancer female general populations, leakage often decreases quality of life. When leakage is treated, quality of life improves [42].

We adopted the hierarchical step-model for causation of bias [43] to review our methodology to limit the risk of confounding factors that may hide an actual association between the variables studied when no real association between them exists. The relationship between the independent variable amount of fecal leakage and the dependent variable frequency of physical activity was still valid after adding the other independent variables to the analysis, which were selected based on previous data on potential moderators of physical activity [10, 29, 35–38]. Regarding misrepresentation, non-participation may induce selection-induced bias. Since we have no information on the non-participating survivors, a strength of our study is that the response rate was 85%. The third step of the hierarchical step-model [43] covers bias induced by misclassification due to incorrect data. A fundamental part of our study was thus the validity of the clinimetric [44] study questionnaire, which was developed according to established [24, 25] previously described methodology [45]. Self-reported questions are the most common and cost-effective measurement of physical activity that can be used in large samples [46]. The risk of recall bias and over and underestimation of physical activity would have been eliminated using accelerometery [46, 47]. However, we found that using accelerometery induces too great a burden on the patient [46, 47] and we therefore adopted self-reported physical activity question. We based the cut-off for categorizing the variable on our knowing that physical activity guidelines propose weekly physical activity [10]. However, a limitation is that we registered only the frequency of moderate level physical activity, not the duration. We thus do not know if the female pelvic cancer survivors who practiced physical activity at least weekly adhered to physical activity guidelines for cancer survivors [10, 11]. However, we clearly know that they practiced physical activity more frequently than the women practicing physical activity less than weekly. Our methodology to collect data in a limited duration of calendar time but with varying length of follow-up time gives us the possibility to depict the trajectory of late effects (manifestations of treatment-induced cancer survivorship diseases and states) without the measurement-induced problems (bias) that may happen when a certain manifestation repeatedly is measured in the same individual. Moreover, we avoid the increase over time in attrition that by and large always happens when the same individuals are followed over time. When we do not stratify according to length of follow-up, we calculate a weighed value for 6–48 months of follow-up. The women received the study questionnaire by postal mail and answered it in privacy, which reasonably lowers the risk of potential therapist-induced bias. Ordinal data were analyzed by methods appropriate for the ordinal and category nature of the data [48]. We did not include the variables quality of life, depressed mood, and anxious mood in the analysis as possibly explaining the variation in physical activity since we do not know the direction of the relationship between quality of life, depressed mood, and physical activity. We selected only characteristics that reasonably could not be consequences of physical activity.

In conclusion, the survivors practicing weekly physical activity experienced better QoL and experienced depressed mood less frequently than the others. Our results indicate that it is important for cancer care professionals to provide extra support to subgroups of pelvic cancer survivors who practice less physical activity, to help them to maintain the best possible quality of life, and to lower the frequency of depressed mood. We found a covariation between physical activity and fecal leakage. And a clear similar trend was seen among those experiencing urinary leakage. We are uncertain to what extent the leakage leads to reduced physical activity and to what extent (in the other way) physical activity can be correlated with muscular exercise of the pelvic floor, which in turn leads to a reduced incidence of fecal leakage or urine leakage. Our results, however, indicate that there is a logical foundation (rationale) to try pelvic floor training in various ways to reduce the occurrence of leakage and thereby increase the possibilities of living a good life.

## Appendix

Study-specific questions regarding physical activity, urinary, and fecal leakage, quality of life, depressed an anxious mood.
